# Seroprevalence and Molecular Detection of Cytomegalovirus UL146 and US28 Gene Expression in Women With Recurrent Pregnancy Loss

**DOI:** 10.7759/cureus.73039

**Published:** 2024-11-05

**Authors:** Niyan Inaam, Samir Othman, Hataw Fryad, Shler Faqi

**Affiliations:** 1 Basic Medical Science, College of Medicine of Hawler Medical University, Erbil, IRQ; 2 Community Medicine, College of Medicine of Hawler Medical University, Erbil, IRQ; 3 Medical Laboratory Science, College of Health Sciences, Lebanese French University, Erbil, IRQ; 4 Basic Medical Science, College of Medicine, University of Sulaimani, Erbil, IRQ

**Keywords:** childbearing age, elisa, hcmv, igg, igm, miscarriage, pcr, stillbirth, ul146, us28

## Abstract

Background

Human cytomegalovirus (CMV) is a global herpesvirus that is highly prevalent worldwide and is able to establish lifelong latency after initial infection. The infection is highly frequent during pregnancy in human beings, which leads to preterm birth in some cases. Circulating strains of CMV carry a high number of variable or disrupted genes. Some of these like *UL146*, a highly diverse gene, and the *US28* gene are involved in viral dissemination. This study aims to determine the seroprevalence of CMV and to investigate whether the highly variable *UL146 *and *US28* genes, isolated from the blood of seropositive women, are associated with recurrent pregnancy loss.

Material and methods

This cross-sectional study was carried out in Erbil City, Iraq from October 2022 to July 2023. A total of 150 women at their reproductive age with a history of miscarriage who attended Maternity Teaching Hospital were enrolled. Anti-CMV IgG and IgM antibodies were assessed by enzyme-linked immunosorbent assay (ELISA). Highly variable *UL146 *and *US28* genes of CMV from seropositive samples were amplified by conventional polymerase chain reaction (PCR), and the results were visualized on a UV-transilluminator. SPSS version 22 (IBM Corp., Armonk, NY, USA) was used for data entry and analysis. The p-value less than 0.05 was regarded as statistically significant.

Results

Anti-CMV IgG and IgM were seropositive in 103 (53.3%) and 13 (8.7%) women, respectively, and only 10 (6.7%) of them for both anti-CMV IgG and IgM. Significant associations of CMV and history of miscarriage, age, educational level, and gestational age of miscarriages were observed (p-value less than 0.05). On the other hand, no statistically significant association between CMV and socioeconomic level or residency was observed. The frequencies of genetic analysis of *UL146* and *US28* of the 103 seropositive tested samples of women with a history of miscarriage were 31 (30.1%) and nine (8.7%), respectively. A significant association between recurrent miscarriage and *UL146* gene expression was observed. PCR targeting the *UL146* demonstrated greater sensitivity for diagnosing CMV.

Conclusion

The seroprevalence of CMV is relatively high in Erbil, and the *UL146 and US28* genes can act as factors in the initial level of CMV. Therefore, molecular detection of these genes can aid in determining the virulence of CMV strains.

## Introduction

The human cytomegalovirus (CMV) or human herpesvirus five is a highly prevalent herpesvirus worldwide, according to the World Health Organization (WHO). CMV is an opportunistic virus that affects people of all ages, and by the age of five, around one in three children in the United States is infected [1]. CMV infection was relatively common among women of reproductive age, with prevalence ranging from 45% to 95% [2]. It is a major source of prenatal and perinatal infections and may cause significant complications in pregnancy [1]. Its clinical presences range from asymptomatic forms (90% of cases) to severe fetal impairment and, in rare cases, death attributed to abortion [3]. Most infection occurrences of CMV are asymptomatic in a host with a normal immune system and usually do not cause any serious consequences. However, maternal CMV infection during pregnancy can pose severe and permanent complications for the fetus [4]. Dissemination of CMV in the blood is an important aspect in the pathogenesis of disease in both primary infection and reactivation. It has been testified that the risk of fetal impairment is greater if the primary infection arises during the first trimester of pregnancy [3]. The seroprevalence of CMV among women of childbirth age ranges from 35% to 95% in different countries, as well as increasing with age [5]. CMV infection during pregnancy is one of the leading causes of congenital infections worldwide, with an incidence of 0.2%-2.2% of live births [6,7]. Up to 15% of such children have neurologic impairment, including delayed development, mental blockage, and neurosensory hearing deficiency [8]. Fetal or neonatal death occurs in about 10% of fetuses or newborns with consequential intrauterine CMV infection [9]. Globally, the seroprevalence of CMV in people of reproductive age is 86% [5].

The seroprevalence of human CMV in developing countries and in societies with lower socioeconomic status is higher than that in developed countries. [10]. Despite the extensive understanding of the pathophysiology and epidemiology of CMV infections in pregnant women over the past few decades, the infection remains largely unknown to most women, and only a limited number of pregnant women are routinely screened for CMV infections during pregnancy. This is due to the fact that, in the majority of countries, no guidelines recommend routine serological screening for CMV during pregnancy [4]. Routine serological screening of pregnant women infected with CMV in developing countries has supported their understanding of CMV infections among pregnant women. This reflection is vital for congenital CMV epidemiology. The rate of congenital infection is directly associated with the seroprevalence of human CMV antibodies in the population [11]. Women of childbearing age are at major risk of giving birth to newborns with congenital infection if the infection is acquired during pregnancy [12]. Seventy percent of miscarriages are linked to human CMV, which can cause a wide range of clinical manifestations in immunocompromised individuals, from severe illness to death. These include congenital infection of fetuses, perinatal infection in neonates, non-hereditary sensorineural hearing loss in offspring (SNHL), organ and bone marrow transplant, HIV, and immunosuppressive patients [13]. Many studies conducted over the years have confirmed the link between CMV infection and abortion during pregnancy. These studies suggest that up to 90% of miscarriages or birth abnormalities can be attributed to CMV recurrence during pregnancy [13-15].

The monopartite, linear, double-stranded DNA genome of CMV is approximately 235 kb in size. More than 750 translated open reading frames (ORFs) are present, and they are separated into two areas by terminal and internal inverted repeats: the unique long (UL) and unique short (US) regions [16]. CMV has adopted a wide range of approaches to avoid immune recognition and facilitate the dissemination of infection, immune evasion, and the creation of latent infection [16,17]. One of the CMV genes is highly inconstant such as the chemokine homolog *UL146* where 14 distinct genotypes have been recognized and novel drug target *US28 *has a variety of numerous N-terminal polymorphisms. CMV uses the *UL146* gene product expressed in infected endothelial cells to attract neutrophils by activating their CXCR1 and CXCR2 receptors, whereby neutrophils can act as carriers of the virus to uninfected endothelial cells. In this way, a lasting pool of CMV-infected endothelial cells could be maintained [17]. The *US28* gene may provide a potential target for therapeutic intervention [16]. Screening for the CMV virus during pregnancy is beneficial, as it allows for the detection of potential fetal infections, thereby enabling the prevention of adverse consequences, such as the birth of a child with physical and mental disabilities. Identifying a viral marker for predicting disease outcomes could have a major impact on prenatal diagnosis [18]. Laboratory tests are the top and only way to diagnose the CMV. In spite of its advantages, amniocentesis is an invasive technique that increases the risk of abortion; consequently, serological and molecular methods are of excessive importance in the timely diagnosis of CMV infection [14,19]. The study aims to analyze the seroprevalence of CMV in women with a history of miscarriage. Virulence and genetic study of highly variable genes, namely *UL146* and *US28,* and their role as risk factors for miscarriage in aborted women.

## Materials and methods

Study design, setting, and duration

This cross-sectional study was carried out in Maternity Teaching Hospital in Erbil, Iraq over the period from October 2022 to July 2023.

Subjects

A convenience sample of 150 pregnant women with a history of primary and recurrent miscarriage was included in this study. Their ages ranged from 18 to 48 years, and all attended the emergency unit of the Maternity Teaching Hospital in Erbil City. All patient personal information, including name and address, is kept private. The miscarriage participants were in the first, second, and third trimesters of pregnancy. The miscarriage participants were in the first, second, and third trimesters of pregnancy. Participants with congenital uterine malformations, cervical incompetence, autoimmune diseases such as rheumatoid arthritis (RA) and systemic lupus erythematosus (SLE), suspected urinary tract infections (UTI) indicated by vaginal discharge, burning during urination, and dysuria, as well as women with obesity, diabetes, molar pregnancy, and those who had induced abortion were excluded from the study.

Design of Questionnaire and Data Collection

After obtaining verbal consent from the participants. A close-ended questionnaire was used for the collection of information from the participants. An informative close-end questionnaire including age, residency, socioeconomic level, history of miscarriage, and current and past obstetrical history was filled out through direct interviews.

Collection of Clinical Specimens

A volume of 5 mL of blood samples was withdrawn aseptically through venipuncture using a disposable plastic syringe and dispensed into two clean sterile screw plastic test tubes. One tube contains 3 mL without anticoagulant (EDTA). The anticoagulant-free samples were centrifuged at 3000 rpm for 10 minutes. The harvested serum was dispensed into two clean Eppendorf tubes (1.5 mL capacity) and stored at -20°C for six months till used for serological detection of CMV by enzyme-linked immunosorbent assay (ELISA). The procedure was done according to the manufacturer’s instructions supplied with the kits (Bioactive diagnostic) by Bioactive Company (Germany). Anticoagulants containing 2 mL blood samples were stored at -40°C and used later after serological analysis for extraction of DNA and molecular detection of CMV according to the manufacturer's instructions. PAX gene blood DNA tubes are used for DNA extraction. 

Detection of Anti-CMV IgM and IgG Antibodies by ELISA

Anti-CMV IgG and IgM antibodies were detected in the sera of women using commercially available quantitative ELISA kits (Bioactive diagnostic) Bioactive Company (Germany), catalog number (CMVG0110BA) IgG, and (CMVM0110BA) IgM in accordance with the manufacturer’s instructions.

Molecular Detection of CMV UL146 and US28 Genes

Isolation of CMV genomic DNA: Two hundred micro litter of genomic DNA were extracted from blood samples with the use of DNA extraction kits (Add-Bio-genomic, Korea), catalog number (10034) according to manufacturer’s instructions.

Genomic DNA extraction procedure: A volume of 20 µL of proteinase solution was added to a 1.5 mL capacity micro-centrifuge tube. A volume of 200 µL of blood sample was transferred to the micro-centrifuge tube, then 200 µL of buffer GB was added to the sample, and then all were mixed by pulse vortexing for 15 seconds and incubated for 10 minutes at 56°C. A volume of 200 µL absolute ethanol was added and mixed well by pulse vortexing for 15 seconds, then the lysate was carefully transferred into the upper reservoir of the spin column (fit in a 2 mL tube) without wetting the edge, and centrifuged at 10000 rpm for 1 min. The spin column was transferred to a new 2 mL collection tube for filtration, after that 500 mL of the buffer GW1 was added to the spin column, and centrifuged at 10000 rpm for 1 min. Flow through was discarded, and the spin column was transferred to a new 2 mL collection tube following centrifugation. A volume of 500 µL of buffer GW2 was added to the spin column, and centrifuged at 10000 rpm for 1 min. After centrifugation, the flow through was discarded and the spin column was reassembled with its collection tube. Centrifuged once more at 12000 rpm for 1-2 min to completely remove ethanol, and ensured that there was no droplet clinging to the bottom of the collection tube. Then the spin column was transferred to a new 1.5 mL tube (not provided) for elution, with 200 µL of buffer GE onto the spin column, then left for 1 min at room temperature. DNA was eluted by centrifugation at 10000 rpm for 1 min. The purity of the yielded DNA was measured by NanoDrop.

Isolation of CMV genomic extraction: The concentration and the purity of the extracted genomic material were estimated using NanoDrop (Thermo Scientific, USA). Absorbance was measured at 260 nm and 280 nm wavelengths to provide an estimation of DNA purity. An absorbance quotient value of 1.8<R<2.0 is considered to be sufficiently pure DNA.

Polymerase chain reaction program and condition: Extracted DNA from 103 seropositive individuals with a history of miscarriage was tested by polymerase chain reaction (PCR) for the detection of *UL146* and *US28* genes, using primers from Berg et al. (2019) as a reference, and was rechecked using the cited reference [16]. The 722-936 base pair fragments of the *UL146* gene and the 387 base pair fragments of the *US28* gene were amplified after identification using the following specific primers, respectively: *UL146*: (F) CCGGGAATACCGGATATTACG and (R) CAGCACTTCCTGACGATTG; *US28*: (F) CCGCTCATATAGACCAAACC and (R) AGGGAGTTGTGATCTAGGAG [16]. Each PCR amplification was carried out in a 25 µL solution consisting of a 12.5 µL master mix (Promega, Korea) composed of Taq DNA polymerase, reaction buffer (pH=8.5), 400 µM dATP, 400 µM dGTP, 400 µM dCTP, 400 µM dTTP, and 3 mM MgCl2. 2 µL (20 ng) of the DNA sample, 1 µL (10 pmL) of each forward and reverse primers and then completed with nuclease-free water to 25 µL. The process of PCR was performed by a PCR Thermal cycler (Corbett Life Science Pty., Ltd. Germany). Initial denaturation was done at 94°C for 90 seconds, followed by 40 cycles of final denaturation at 94°C for 50 seconds. The annealing phase was carried out at 64°C for 50 seconds, and the extension was performed at 72°C for 50 seconds. The final extension was also performed at 72°C for 10 minutes. The PCR product was then run on a 1.5% agarose gel and stained with 0.2 mg/mL safe dye (Add-Bio, South Korea) for visualization under ultraviolet light (Cleaver Scientific., Thistle Scientific., UK). A 100 bp ladder (Promega Corp., Madison, Wisconsin, USA) was used as a marker.

Ethical considerations

The study was approved by the Research Ethics Committee of the College of Medicine, Hawler Medical University, Erbil, Iraq issued approval Meeting code 7, Paper code 12, dated: 27 May 2022. A verbal consent was obtained from each participant before the collection of the sample.

Statistical analysis

Data were analyzed using SPSS software program version 22 (IBM Corp., Armonk, NY). Results were expressed as frequencies, percentages, Chi-square, and Fisher’s exact test were used to determine any significant difference between the categorical data. The p-value<0.05 was regarded as statistically significant.

## Results

**Figure 1:**
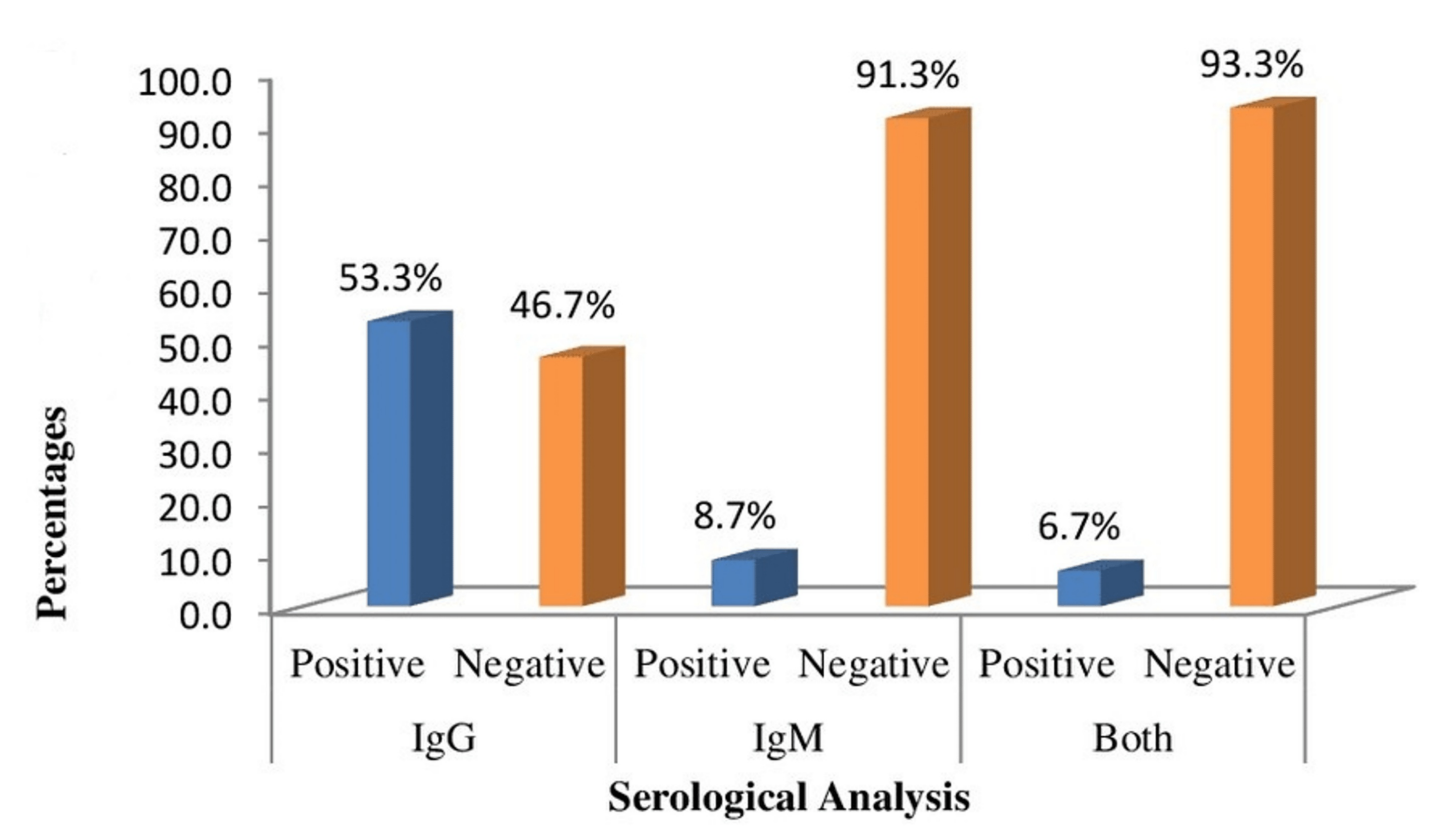
Seropositivity of anti-CMV IgG and IgM antibodies among 150 miscarriage women in Erbil City Prevalence of anti-CMV IgG and IgM antibodies among 150 miscarriage women in Erbil City. CMV: cytomegalovirus

A total of 150 women agreed to be involved in this study. Three-quarters of the participants 103 (68.7%) had antibodies for CMV. Anti-CMV IgG and IgM were seropositive in 80 (53.3%) and 13 (8.7%) women, respectively. Only 10 (6.7%) of women who were seropositive carried both anti-CMV IgG and IgM as expressed in Figure 1.

As shown in Table 1, regarding certain socio-demographic characteristics of the studied participants, the results revealed a statistically non-significant highest rate of anti-CMV IgG, IgM, and both IgG and IgM among women with low socioeconomic status, as well as the highest rates of IgG and both IgG and IgM in urban inhabitants. On the other hand, a statistically significant association (p<0.05) was found between the educational level of the participants with CMV. This study also revealed statistically significant differences (p<0.05) in the levels of both IgG and IgM antibodies among the studied age groups.

**Table 1:** Seropositivity of CMV among 150 miscarriage women according to certain socio-demographic characteristics of the studied participants ^*^Calculated using Fisher’s Exact test and p-value is calculated using Chi-square. CMV: cytomegalovirus

Variables	No. of the examined sample	IgG no. (%) seropositive samples	Chi-Square value, p-value	IgM no (%) seropositive samples	Chi-Square value, p-value	IgG & IgM no. (%) seropositive samples	Chi-Square value, p-value
Socioeconomic level							
Below sufficient	92	50 (33.3)	0.920*	10 (6.7)	*0.587	9 (6.0)	0.180*
Sufficient	51	26 (17.3)		3 (2.0)		1 (0.7)	
Above sufficient	7	4 (2.7)		0 (0.0)		0 (0.0)	
Educational level							
Primary	32	11 (7.3)	8.329, 0.039	1 (0.7)	0.520*	1 (0.7)	0.161*
Secondary	32	15 (10.0)		2 (1.3)		0 (0.0)	
University	20	12 (8)		2 (1.3)		2 (1.3)	
Illiterate	66	42 (28)		8 (5.4)		7 (4.7)	
Residency							
Urban	80	48 (32.0)	3.061, 0.080	6 (4.0)	0.295, 0.587	6 (4.0)	0.751*
Rural	70	32 (21.3)		7 (4.7%)		4 (2.7%)	
Age							
≤20	17	9 (6.0)	0.365*	1 (0.7)	0.089*	0 (0.0)	0.036*
21-30	80	40 (26.7)		4 (2.7)		3 (2.0)	
31-40	46	25 (16.6)		6 (4.0)		5 (3.4)	
≥41	7	6 (4.0)		2 (1.3)		2 (1.3)	
Total	150	80 (53.3)		13 (8.7)		10 (6.7)	

Regarding gynecological observation, a statistically significant association (P<0.05) of CMVs with history of miscarriage was observed on screening for both anti-CMV IgG and IgM antibodies 8 (5.4%) versus 2 (1.3%) by ELISA, respectively, while a statistically non-significant (p>0.05) association were observed in the distribution of anti-CMV IgG, IgM, both IgG and IgM Abs among gestational age, as revealed in Table 2

**Table 2:** Seropositivity of anti-CMV antibodies according to the history of miscarriage and gestational age among studied participants ^*^Calculated using Fisher’s Exact test and p-value is calculated using Chi-square. CMV: cytomegalovirus

Variables	No. of the examined sample	IgG no (%) seropositive samples	Chi-square value, p-value	IgM no (%) seropositive samples	Chi-square value, p-value	IgG & IgM no (%) seropositive samples	Chi-square value, p-value
History of miscarriage							
Primary	70	36 (24.0)	0.191, 0.662	9 (6.0)	2.912, 0.088	8 (5.4)	0.030*
Recurrent	80	44 (29.3)		4 (2.7)		2 (1.3)	
Gestational age							
First trimester	50	24 (16.0)	0.368	7 (4.7)	0.169*	7 (4.7)	0.062*
Second trimester	90	52 (34.6)		5 (3.3)		3 (2.0)	
Third trimester	10	4 (2.7)		1 (0.7)		0 (0.0)	
Total	150	80 (53.3)		13 (8.7)		10 (6.7)	

**Figure 2:**
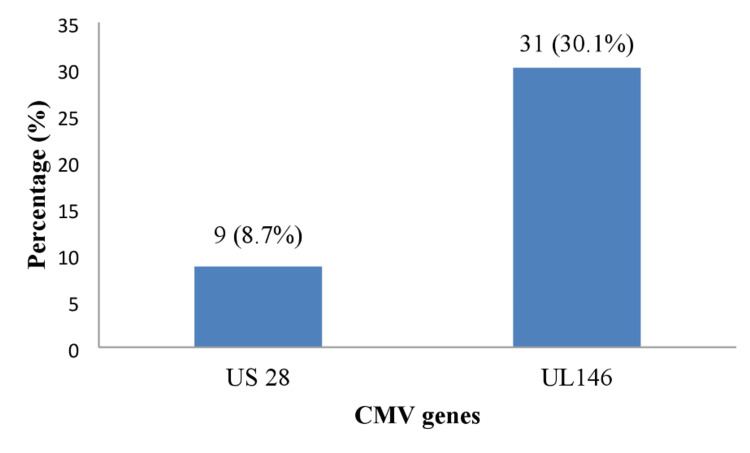
The percentage of CMV highly variable genes UL146 and US28 in 103 seropositive samples by PCR PCR: polymerase chain reaction; CMV: cytomegalovirus

A total of 103 seropositive samples were tested by conventional PCR for the detection of *UL146* and *US28* genes by primers. About 30.1% and 8.7% of them showed positive results for *UL146* and *US28,* respectively. PCR targeting the *UL146* gene was more efficient as shown in Figure 2.

Table 3 shows the results of molecular detection of CMV in the blood of women with a history of miscarriage, which showed seropositive results for anti-CMV IgM and IgG by ELISA. There was a significant association between *UL146 *(p<0.001) and a history of miscarriage. While there were no differences between women with primary miscarriage and those with recurrent miscarriages, in regard to *US28*, PCR targeting both CMV genes detected more recurrent miscarriage cases than primary miscarriage among anti-CMV seropositive women.

**Table 3:** Molecular detection of UL146 and US28 genes of CMV among 103 seropositive samples by PCR in terms of history of miscarriage ^*^Calculated using Fisher’s Exact test and p-value is calculated using Chi-square. PCR: polymerase chain reaction; CMV: cytomegalovirus

History of miscarriage	No. of examined samples	UL146	Chi-square value, p-value	US28	Chi-square value, p-value
		Positive samples no. (%)		Positive samples no. (%)	
Primary	50	24 (23.3%)	14.803, <0.001	7 (6.8%)	0.087*
Recurrent	53	7 (6.8%)		2 (1.9%)	
Total	103	31 (30.1%)		9 (8.7%)	

**Figure 3:**
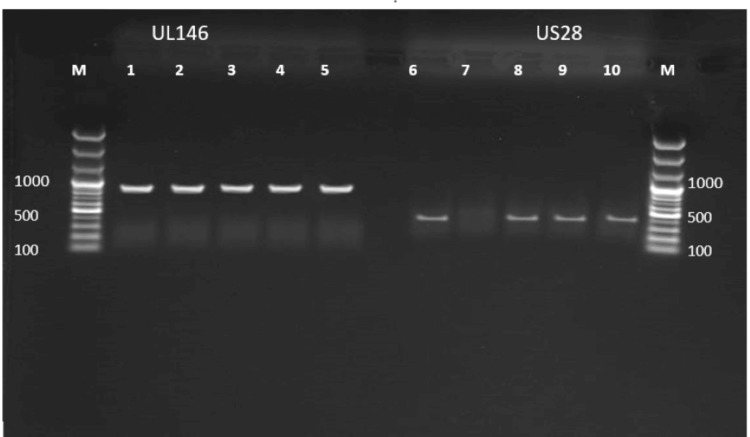
Gel electrophorese shows UL146 and US28 genes of CMV among seropositive samples by PCR in terms of the history of miscarriage PCR amplification of the *UL146* and *US28* genes of the CMV genome on a 1.5% agarose gel stained with a safe dye. PCR: polymerase chain reaction; CMV: cytomegalovirus

PCR amplification of the *UL146* and *US28* genes of the CMV genome was performed on a 1.5% agarose gel stained with a safe dye using primers specific for CMV. Positive samples revealed 850 bp bands for the *UL146* gene and 387 bp bands for the *US28* gene. M: DNA marker (100 bp). Lanes 1 to 5 contained positive samples for *UL146*, and Lanes 6, 8, 9, and 10 contained positive samples for the *US28* gene as shown in Figure 3.

## Discussion

CMV is an enveloped DNA virus, like other members of the herpes virus family, that develops lifelong latency following primary infection and resides in monocytes and granulocytes. Consequently, vertical transmission may occur via original infection, illness recurrence, or contamination with another strain [20]. The severity of the infection depends on the gestational age at which the infection has occurred. If it occurred during the early stages, the transfer rate is low but the severity is high if the fetus is infected; if it occurred during the later stages, the transmission rate is higher but the severity is low [13].

The recognition of CMV-specific antibodies is the primary diagnostic process to determine infection with CMV. Serologic tests are used to detect acute infection in pregnant women, but false-positive results occur frequently. Therefore, serologic diagnosis must be established at a reference laboratory before treatment with potentially toxic drugs is considered [11].

In the present study, serological screening of CMV among 150 pregnant women with a history of miscarriage revealed that 80 (53.3%) of the samples were seropositive for IgG, 13 (8.7%) for IgM, and 10 of the cases (6.7%) were positive for both IgG and IgM. This finding was similar to that observed by a previous study, carried out in Iraq. The researchers found that the seropositivity rate of CMV was 98% and 6% for IgG and IgM, respectively, among women who were referred to the outpatient clinic at Al-Basrah Gynecology and Obstetrics Hospital, Al-Zubair General Hospital, and Dar Al-Shifa Investment Hospital during the period from February 2020 to April 2021 in Basrah city, Iraq [13]. 

CMV is one of the causes of miscarriage in pregnant women. Most cases of miscarriage occur in the acute phase of infection and early pregnancy [21]. In the present study, patients with recurrent pregnancy loss were significantly more seropositive than those with primary infection, suggesting that previous exposure to CMV might be a risk factor for recurrent pregnancy loss. This result is in line with the results of a previous study that found an association between spontaneous abortion and the presence of a latent CMV infection [22]. In the present study, the socio-demographical characteristics of the participants with higher seropositivity for CMV were characterized by more years of life, fewer years of study, and lower family income, which are all potential risk factors for CMV infection. Socioeconomic factors have also been related to the risk of developing the congenital form of CMV, these factors interfere directly with the quality of prenatal care. Women of low socioeconomic status may be at risk for repeated infections due to the unhygienic environments in which they reside. The majority of the studies reviewed reported a significant association between CMV and these risk factors [23,24]. Nevertheless, in the present study, no significant association between anti-CMV antibodies and socioeconomic level was observed. Regarding the age of the participants, the current study observed the highest rate of anti-CMV IgG antibodies, with 40 (26.7%) found among women aged 21 to 30 years. However, statistical analysis revealed no significant association. A study that was made in Sudan estimated that the seroprevalence of CMV increased in ages between 25 and 34 years old; this is due to women at this age being at high risk and the most fertile period of childbearing age [19,25]. In Bangladesh, the highest rate of CMV was observed in women of high reproductive age (>20 years old), likely because they are more engaged in various home-based activities after marriage, which increases their risk of exposure to the infection [26].

The present findings revealed statistical differences in educational level (p>0.05) among pregnant women. This result was in accordance with that obtained by Zenebe and coworkers (2021) in Ethiopia, who showed that an effective method of avoiding infection in pregnant women is education [27]. In the present study, the highest rates of CMV, 48 (32%) for IgG and six (4%) for both IgG and IgM, were observed among women who were urban inhabitants. The results of the current study are similar to those of a study conducted among residents in Southern Nigeria, which found that the prevalence of human CMV infection was higher in urban areas (30, 61.2%) than in rural communities (19, 38.8%) [28]. The high levels of HCMV infection in urban areas rather than rural areas may be due to a huge increase in urban population triggering basic infrastructure to be insufficient, coupled with social and economic discriminations. Higher rates of social collaboration, viable sex activity, several sexual partners, socioeconomic standards, and racial variances between the populations are effective factors that might be responsible for this distinction in prevalence rates [25]. Women who may get an infection during pregnancy may show a variety of clinical signs and symptoms depending on numerous factors, such as the strength of the infection, the virulence of the strain, and the period during which the mother attained the infection [22]. If the mother is infected in the first trimester, it will result in miscarriages, stillbirths, or severe disease of the fetus [11]. One of the main causes of birth abnormalities, which can range from developmental issues to stillbirth, is congenital CMV infection. While the majority of CMV-affected neonates do not exhibit symptoms at birth, they are at risk for sequelae in later childhood, which occurs in many of the cases [4]. The seropositivity of anti-CMV Abs found in the present study did not show the presence of any significant differences (p>0.05) between gestational age (trimesters) of the fetus and seropositivity, but analysis of the results based on gestation age revealed the highest seroprevalence among aborted women who were at their second trimester. The present study aligns with the study by Aliyu et al. (2024) in Nigeria, which also did not find any significant association between CMV infection and gestational age. They reported the highest seropositivity in the second trimester, which was 35.6% [29].

There is a significant genetic difference between the circulating strains of CMV, with 75% of the strains having disruptive mutations and polymorphisms in many genes, according to a recent study [16]. Molecular detection of CMV by PCR revealed variable reactions. Out of 103 samples of miscarriage women tested by PCR using specific primers targeting *UL146* and *US28* genes 31 (30.1%) and nine (8.7%) of the samples showed positive results for *UL146* and *US28*, respectively. Using the *UL146* gene as a target for PCR seems to be more efficient for molecular detection of CMV. Although the sequence of the human CMV genome is commonly well-preserved among clinical strains, some ORFs are highly inconstant. *UL146*, which encodes CXC chemokine homologs, is among these variable ORFs, and the* US28*-encoded chemokine receptor plays an important role in the dissemination of latent human CMV [16,17]. In the present study, a higher number of samples with positive *UL146* and *US28 *genes were detected among women with primary miscarriage, with 24 (23.3%) for *UL146* and seven (6.8%) for *US28*, compared to seven (6.8%) for *UL146* and two (1.9%) for *US28* in women with a history of recurrent miscarriage. Statistically, a significant difference (p<0.001) in *UL146* was detected. The present study is in line with that observed by Guo, et al. (2017) who found that the *UL146* genotype was significantly associated with CMV infection [30]. It has been reported by Berg and Coinvestigator (2019) that* UL146* has been identiﬁed as an important virulence factor for CMV and plays a role during the primary infection in humans in addition to that *UL146* serves as a central regulator of virus duplication and spread at early phase of infection [16]. Regarding the *US28* gene, it is clear that *US28* is necessary for human CMV latency and may provide a potential target for therapeutic involvement. The CMB has evolved a variety of defense mechanisms to avoid immune recognition and promote infection spread. These tactics rely on controlling and reversing the host's immune reaction during infection, for example, by expressing homologs of receptors and ligands encoded by viruses that are crucial for the proper operation of the human immune system. Compared to other human herpesviruses, it encodes two to three times as many gene products, many of which have been demonstrated to interact with and influence the human immune system [16,17].

The strengths and limitations of the study

The strength is that this study was designed to determine the prevalence of CMV and the genetic bases of two highly variable genes of CMV in miscarriage women in Erbil City. These two genes are necessary for human CMV latency, and they facilitate dissemination of infection. To our knowledge, this is the first study to be carried out to study two genes namely *UL146* and *US28,* and their role as factors for primary and recurrent miscarriage in pregnant women in Erbil City, Iraq. The limitations of the study include its cross-sectional design and the fact that it was conducted in a single major maternity hospital in Erbil City, which primarily serves women from specific socioeconomic backgrounds. Women from higher social classes might be underrepresented, as they may prefer private healthcare facilities. However, this effect might be small, given that this hospital is a major healthcare provider in the region and serves a broad spectrum of the population.

## Conclusions

The study concluded that the seropositivity of CMV is relatively high among women who have experienced miscarriages in Erbil City. A significant association between CMV and a history of miscarriage was observed in women who were seropositive for both anti-CMV IgG and IgM antibodies. Parameters of socioeconomic level and residency affect CMV infection. On the other hand, age and education parameters have no effect on CMV infection. The highly variable *UL146* gene is an efficient target for PCR; it avoids immune recognition and facilitates the dissemination of infection, immune evasion, and the establishment of latent infection, and it could serve as a factor in abnormal pregnancy outcomes.
